# Prior exposure to corticosterone markedly enhances and prolongs the neuroinflammatory response to systemic challenge with LPS

**DOI:** 10.1371/journal.pone.0190546

**Published:** 2018-01-05

**Authors:** Kimberly A. Kelly, Lindsay T. Michalovicz, Julie V. Miller, Vincent Castranova, Diane B. Miller, James P. O’Callaghan

**Affiliations:** 1 Centers for Disease Control and Prevention, National Institute for Occupational Safety and Health, Morgantown, West Virginia; 2 Department of Pharmaceutical Sciences, School of Pharmacy, West Virginia University, Morgantown, West Virginia; Universidade de Sao Paulo, BRAZIL

## Abstract

Systemic exposure to the inflammagen and bacterial endotoxin lipopolysaccharide (LPS) has been widely used to evaluate inflammation and sickness behavior. While many inflammatory conditions occur in the periphery, it is well established that peripheral inflammation can affect the brain. Neuroinflammation, the elaboration of proinflammatory mediators in the CNS, commonly is associated with behavioral symptoms (e.g., lethargy, anhedonia, anorexia, depression, etc.) termed sickness behavior. Stressors have been shown to interact with and alter neuroinflammatory responses and associated behaviors. Here, we examined the effects of the stress hormone, corticosterone (CORT), as a stressor mimic, on neuroinflammation induced with a single injection (2mg/kg, s.c.) or inhalation exposure (7.5 μg/m^3^) of LPS or polyinosinic:polycytidylic acid (PIC; 12mg/kg, i.p.) in adult male C57BL/6J mice. CORT was given in the drinking water (200 mg/L) for 1 week or every other week for 90 days followed by LPS. Proinflammatory cytokine expression (TNFα, IL-6, CCL2, IL-1β, LIF, and OSM) was measured by qPCR. The activation of the neuroinflammation downstream signaling activator, STAT3, was assessed by immunoblot of pSTAT3^Tyr705^. The presence of astrogliosis was assessed by immunoassay of GFAP. Acute exposure to LPS caused brain-wide neuroinflammation without producing astrogliosis; exposure to CORT for 1 week caused marked exacerbation of the LPS-induced neuroinflammation. This neuroinflammatory “priming” by CORT was so pronounced that sub-neuroinflammatory exposures by inhalation instigated neuroinflammation when paired with prior CORT exposure. This effect also was extended to another common inflammagen, PIC (a viral mimic). Furthermore, a single week of CORT exposure maintained the potential for priming for 30 days, while intermittent exposure to CORT for up to 90 days synergistically primed the LPS-induced neuroinflammatory response. These findings highlight the possibility for an isolated inflammatory event to be exacerbated by a temporally distant stressful stimulus and demonstrates the potential for recurrent stress to greatly aggravate chronic inflammatory disorders.

## Introduction

Inflammation can result from acute exposures to pathogens, chemicals, physical damage, irritants, and chronic illnesses. As a component of the innate immune response, inflammation is characterized by the activation of macrophages and their subsequent release of cytokines. While many inflammatory conditions occur in the periphery, it has been well established that peripheral inflammation can exert effects on the brain, as reflected in the enhanced expression of proinflammatory mediators. Anti-inflammatory compounds can ameliorate these neuroinflammatory responses, as well as systemic inflammation. For example, therapy with the classic anti-inflammatory rodent glucocorticoid, corticosterone (CORT), can suppress neuroinflammation and neuroinflammatory signaling caused by exposure to neurotoxins or the bacterial endotoxin, lipopolysaccharide (LPS) [1]. Paradoxically, prior treatment with CORT can enhance rather than suppress the neuroinflammatory response to neurotoxicants [2], acetylcholinesterase inhibitors [3,4], and LPS [5,6]. These results suggest that glucocorticoids can either ameliorate or potentiate the response to a neuroinflammagen depending on whether the treatment occurs before or after an inflammatory exposure.

Increases in plasma cortisol in humans or of CORT in rodents result from stressor exposures. Serum CORT levels frequently are used as indicators of a stress response; accordingly, exogenous CORT administration can be used to mimic stressor exposures in rodents. Precedent exists for a proinflammatory effect of stress hormones [7,8]. The influence of stressors on inflammation vary depending on the duration and frequency of the stressor response. Thus, exposure to acute stressors can either be protective or detrimental to the immune system. However, repeated exposure to a stressor has been shown to profoundly exacerbate inflammation [7–10]. Several studies utilizing various stressors (such as restraint, social defeat, forced swim and isolation) in rodent models show exacerbation of the inflammatory response to LPS after stressor exposure [11–16]. These findings suggest that prolonged or repeated elevations of CORT may predispose the CNS to a heightened or prolonged neuroinflammatory response.

In this study, we sought to determine if chronic exposure to CORT could produce persistent, LPS-induced neuroinflammation. While several studies have examined the immediate effects of CORT exposure on neuroinflammation [17–19], here we investigated the effects of a single, week-long exposure to CORT, as well as recurring exposures to CORT over several months, on LPS-induced neuroinflammation. These CORT exposure regimens induce a persistent and escalating “priming” of the neuroinflammatory response to LPS, findings suggestive of a role for repeated exposures to stressors in the development of chronic neuroinflammatory disorders.

## Materials and methods

### Materials

The following drugs and chemicals were provided by or obtained from the sources indicated: LPS from *Esherichia coli* serotype 055:B5 (Cat. Number L2880, Sigma-Aldrich, St. Louis, MO, USA), polyinosinic:polycytidylic acid (PIC; Invivogen, San Diego, CA, USA), CORT (Steraloids, Inc., Newport, RI, USA). The materials used in the glial fibrillary acidic protein (GFAP) assay have been described in detail [20,21]. All other reagents and materials were of at least analytical grade and were obtained from a variety of commercial sources.

### Animals

Male C57BL/6J mice (n = 5 mice per group), 4–6 weeks of age were purchased from Jackson Labs (Bar Harbor, ME, USA). All procedures were performed within protocols approved by the Institutional Animal Care and Use Committee of the Centers for Disease Control and Prevention, National Institute for Occupational Safety and Health, and the animal colony was certified by the Association for Assessment and Accreditation of Laboratory Animal Care. Upon receipt, mice were housed individually in a temperature-controlled (21 ± 1°C) and humidity-controlled (50 ± 10%) colony room maintained under filtered positive-pressure ventilation on a 12h light/12h dark cycle beginning at 0600. The plastic tub cages were 46 cm in length by 25 cm in width by 15 cm in height; cage bedding consisted of heat-treated pine shavings spread at a depth of approximately 4 cm. Food (Harlan 7913 irradiated NIH-31 modified 6% rodent chow) and water were available ad libitum. The mice in this study were individually housed in order to correlate with other previously studies evaluating CORT priming of neuroinflammation [2,3,4].

### Dosing

For short-term exposures, CORT was given in the drinking water (200 mg/L in 0.6% EtOH) for 1 week prior to LPS, PIC, or vehicle treatment on day 8. This regimen of CORT was chosen because of its ability to achieve high circulating levels of this hormone, similar to those achieved with repeated stress [22], and because it was capable of producing significant immunosuppression as evidenced by involution of the thymus (e.g., see O’Callaghan *et al*., 1991 [23]). LPS was administered in one of two ways: 1) injected subcutaneously (s.c.) at 2 mg/kg where vehicle was 0.9% saline; or 2) whole body inhalation exposure [24] to aerosolized LPS (7.5 μg/m^3^)(~7.5 x 10^4^ Endotoxin Units/m^3^) for 3 hours where vehicle was water. PIC was administered intraperitoneally at 12 mg/kg (0.9% saline vehicle). For 30 or 90 day exposures, CORT was given in the drinking water (200 mg/L in 0.6% EtOH) for 1 week prior to LPS or vehicle injection on day 37 or 97, respectively. However, for the 90 day CORT +++ exposure, CORT was given in the drinking water (200 mg/L in 0.6% EtOH) every other week for 13 weeks prior to LPS exposure on day 97. Mice were killed by decapitation or focused microwave irradiation (see below) at 2, 6, 12, or 72 hours post-LPS or vehicle exposure and 6 hours post-PIC exposure.

### Brain dissection and tissue preparation

Immediately after decapitation, whole brains were removed from the skull with the aid of blunt curved forceps. Striatum, hippocampus, cortex, cerebellum, olfactory bulb, and hypothalamus were dissected free hand on a thermoelectric cold plate (Model TCP-2, Aldrich Chemical Co., Milwaukee, WI, USA) using a pair of fine curved forceps (Roboz, Washington, DC, USA). Brain regions from one side of the brain were frozen at -85°C and used for subsequent isolation of total RNA; brain regions from the other side of the brain were used for total and specific protein analysis. These regions were weighed and homogenized with a sonic probe (model XL-2005, Heat Systems, Farmingdale, NY, USA) in 10 volumes of hot (90–95°C) 1% sodium dodecyl sulfate (SDS), and stored frozen at -70°C before total protein assay and immunoassay of GFAP. A separate set of mice were used for phosphorylated signal transducer and activator of transcription 3 at tyrosine 705 (pSTAT3^Tyr705^) analysis and were sacrificed by focused microwave irradiation (Muromachi Kikai, Inc., Tokyo, Japan; Model TMW-4012C, 3.5 KW applied power, 0.90 sec) to preserve steady-state phosphorylation for analysis using phospho-state-specific antibodies [25]. Microwave-fixed brain regions were processed for protein analysis as described above.

### RNA isolation, cDNA synthesis, and real-time PCR amplification

The detailed protocols can be found at protocols.io (DX.DOI.ORG/10.17504/PROTOCOLS.IO.HTRB6M6; DX.DOI.ORG/10.17504/PROTOCOLS.IO.HTVB6N6; DX.DOI.ORG/10.17504/PROTOCOLS.IO.HTWB6PE). Total RNA from striatum, hippocampus, cortex, cerebellum, olfactory bulb, and hypothalamus was isolated using Trizol^®^ reagent (Thermo Fisher Scientific, Waltham, MA, USA) and Phase-lock heavy gel (Eppendorf AG, Hamburg, Germany). The RNA was further cleaned using RNeasy mini spin columns (Qiagen, Valencia, CA, USA). Total RNA (1 μg) was reverse transcribed to cDNA using SuperScript^™^ III and oligo (dT)_12-18_ primers (Thermo Fisher Scientific, Waltham, MA, USA) in a 20 μL reaction. Real-time PCR analysis of the housekeeping gene, glyceraldehyde-3-phosphate dehydrogenase (GAPDH), and of the proinflammatory mediators, TNFα, IL-6, CCL2, IL-1β, leukemia inhibitor factor (LIF), and oncostatin M (OSM) was performed in an ABI7500 Real-Time PCR System (Thermo Fisher Scientific, Waltham, MA, USA) in combination with TaqMan^®^ chemistry. Specific primers and dual-labeled internal fluorogenic (FAM/TAMRA) probe sets (TaqMan^®^ Gene Expression Assays) for these genes were procured from Thermo Fisher Scientific (Waltham, MA, USA) and used according to the manufacturer’s recommendations. All PCR amplifications (40 cycles) were performed in a total volume of 50 μL, containing 1 μL cDNA, 2.5 μL of the specific assay of demand primer/probe mix, and 25 μL of Taqman^®^ Universal master mix. Relative quantification of gene expression was performed using the comparative threshold (ΔΔC_T_) method. Changes in mRNA expression levels were calculated after normalization to GAPDH. The ratios obtained after normalization are expressed as fold change over corresponding saline-treated controls.

### Protein assay

A detailed protocol can be found at protocols.io (DX.DOI.ORG/10.17504/PROTOCOLS.IO.HSWB6FE). Total protein was determined using the Pierce BCA Protein Assay Kit (Thermo Fisher Scientific, Waltham, MA, USA), per the manufacturer’s instructions. Absolute absorbance was measured using the Spectra Max Plus microplate reader and analyzed using Soft Max Pro Plus software (Molecular Devices, Sunnyvale, CA, USA).

### GFAP immunoassay

A detailed protocol can be found at protocols.io (DX.DOI.ORG/10.17504/PROTOCOLS.IO.HSUB6EW). GFAP was assayed in accordance with a previously described procedure [20,21]. In brief, a rabbit polyclonal antibody to GFAP (1:400; DAKO, Carpenteria, CA, USA) was coated on the wells of Immulon-2 microtiter plates (Thermo Labsystems, Franklin, MA). The SDS homogenates and standards were diluted in phosphate-buffered saline (pH 7.4) containing 0.5% Triton X-100. Standards consisted of SDS homogenates of hippocampus with known concentration of GFAP and were prepared the same way as the samples. After blocking non-specific binding with 5% non-fat dry milk, aliquots of the homogenate and standards were added to the wells and incubated. Following washes, a mouse monoclonal antibody to GFAP (1:250; Sigma Chemical Co., St. Louis, MO, USA) was added to ‘sandwich’ the GFAP between the two antibodies. An alkaline phosphatase-conjugated antibody directed against mouse IgG (1:2000; Jackson ImmunoResearch Labs, West Grove, PA, USA) was then added and a colored reaction product was obtained by subsequent addition of the enzyme substrate p-nitrophenol. Quantification was achieved by spectrophotometry of the colored reaction product at 405 nm in the microplate reader (see above), and analyzed with the same software. The amount of GFAP in the samples was calculated as micrograms of GFAP per milligram total protein.

### pSTAT3 immunoblot analysis

A detailed protocol can be found at protocols.io (DX.DOI.ORG/10.17504/PROTOCOLS.IO.HT4B6QW). Activation of the JAK-STAT3 neuroinflammation effector pathway [1] was assessed by quantifying pSTAT3^Tyr705^ from immunoblots with detection of fluorescent signals using an infrared fluorescence scanner (Licor Biosciences; Lincoln, NE, USA) as described previously [1,25–27]. Briefly, following incubation with primary antibodies (rabbit anti-phospho-STAT3^Tyr705^[1:500]; Cell Signaling, Danvers, MA, USA), blots were washed with phosphate buffered saline with 0.1% Tween-20 and incubated with anti-rabbit fluorescent-labeled secondary antibody (1:2500) for 1h prior to scanning by Licor.

### Statistics

Statistical analysis of data was performed utilizing SigmaPlot (v. 11.0). The test of significance was performed using either one-way ANOVA (Saline x LPS) or two-way ANOVA (pretreatment [Water or CORT] x exposure [Saline or LPS/PIC]) on log transformed values followed by multiple pairwise comparison analysis using Fisher least significant difference (LSD) post hoc test with statistical significance at 5% (p<0.05). Graphical representations are mean ± SEM.

## Results

### Systemic exposure to LPS is neuroinflammatory

**Fig 1:**
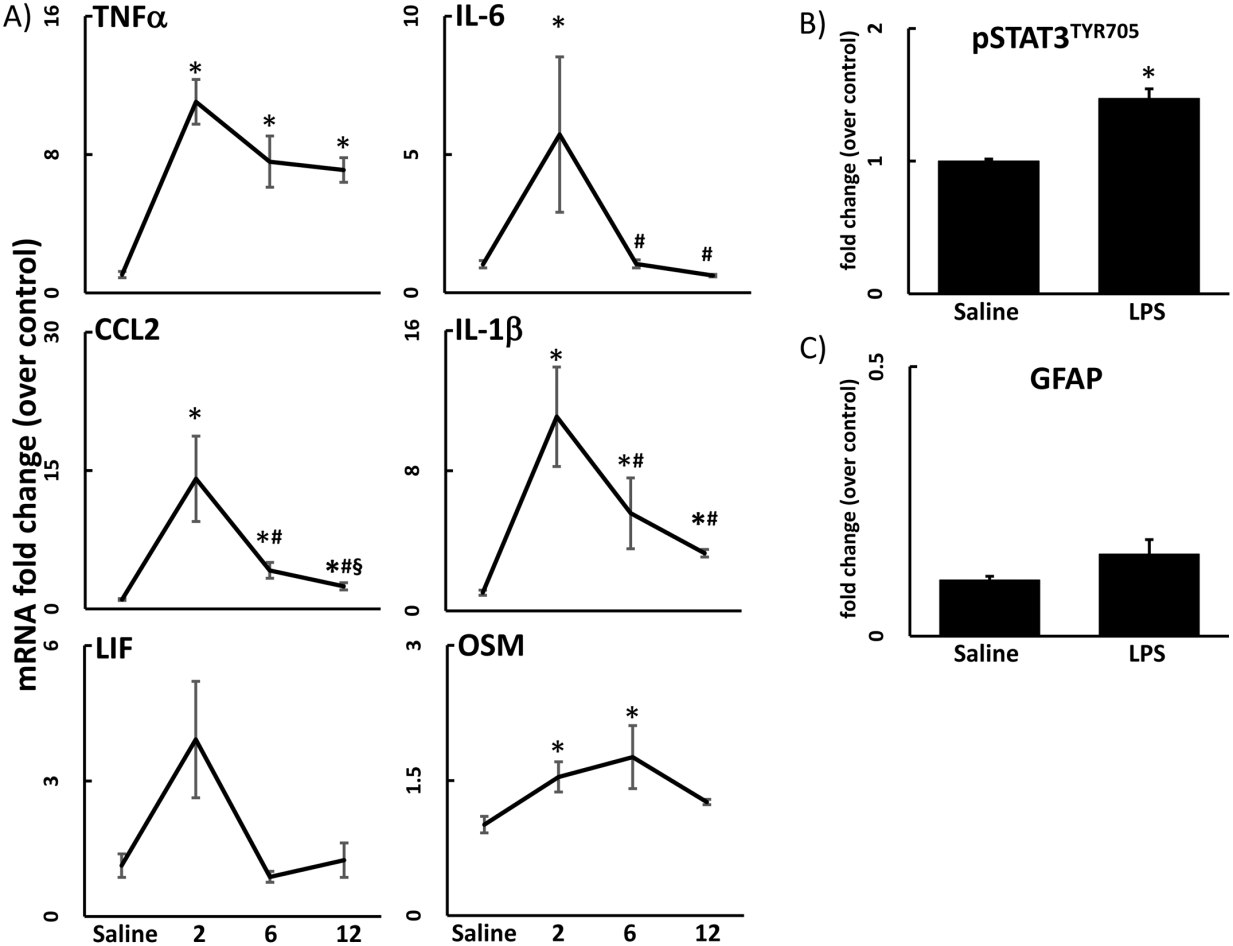
Systemic LPS exposure is neuroinflammatory but does not induce astrogliosis. A) The effects of LPS (2 mg/kg, s.c.) on TNFα, IL-6, CCL2, IL-1β, LIF, and OSM mRNA levels in samples prepared from cortex were determined at 2, 6, and 12 hours after LPS exposure. Data represents mean ± SEM (n = 5 mice/group). Statistical significance of at least p<0.05 is denoted by * as compared to control, # as compared to 2h LPS, and § as compared to 6h LPS. B & C) The effects of LPS exposure (2 mg/kg, s.c.) on activation of the STAT3 signaling module was assessed by immunoblots of pSTAT3^Tyr705^ and astrogliosis was assessed by immunoassay of GFAP. Cortex samples were analyzed for pSTAT3^Tyr705^ (6 hours) and GFAP (72 hours) after LPS exposure. Data represents mean ± SEM (n = 5 mice/group). Statistical significance of at least p<0.05 is denoted by * as compared to control.

**Fig 2:**
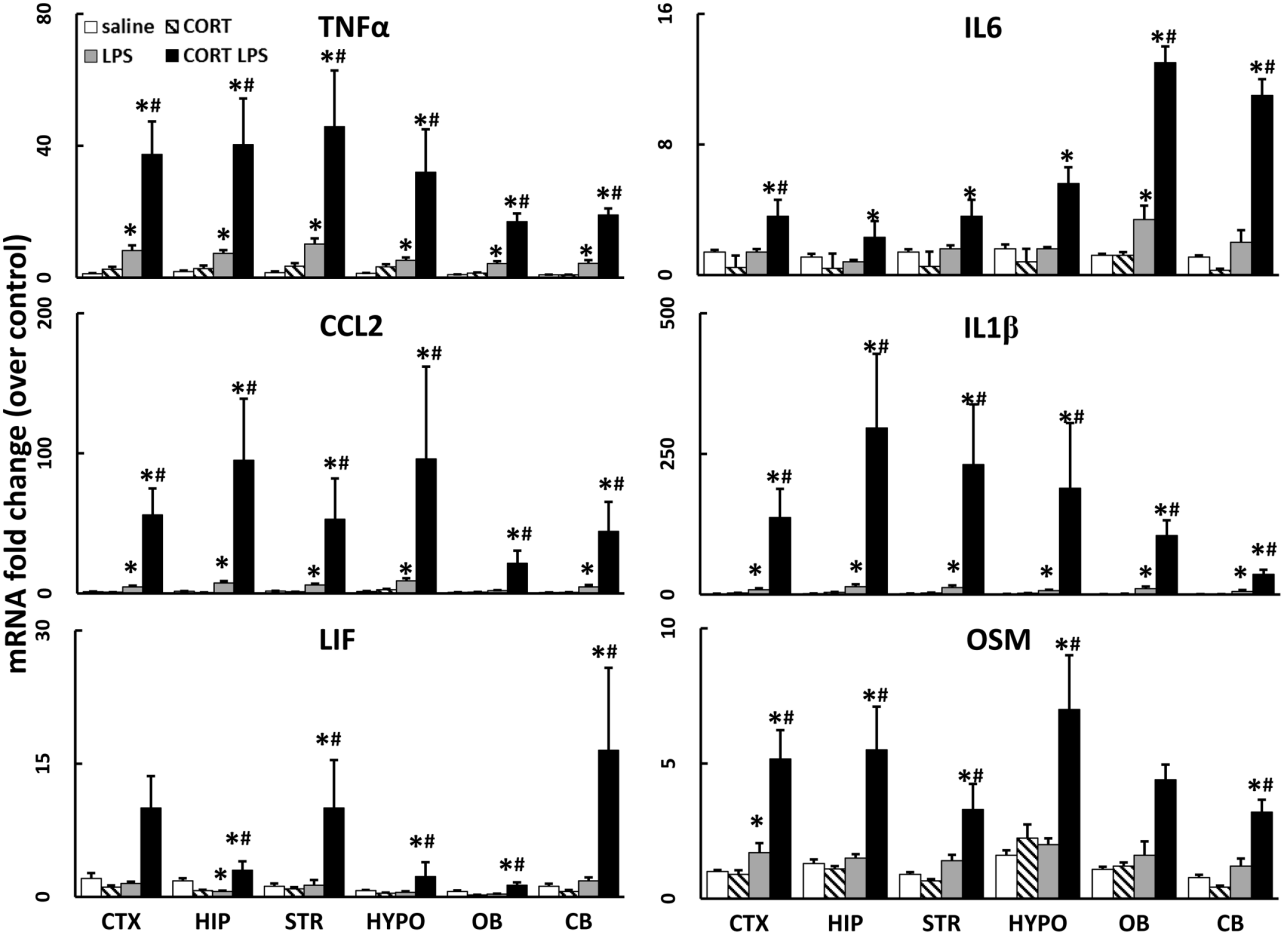
Chronic CORT exacerbates LPS-induced neuroinflammation brain wide. The effects of chronic CORT pretreatment (200 mg/L 0.6% EtOH in drinking water for one week) on LPS (2 mg/kg, s.c.)-induced TNFα, IL-6, CCL2, IL-1β, LIF, and OSM mRNA levels were measured in samples prepared from cortex (CTX), hippocampus (HIP), striatum (STR), hypothalamus (HYPO), olfactory bulb (OB), and cerebellum (CB) 6 hours after LPS exposure. Data represents mean ± SEM (n = 5 mice/group). Statistical significance of at least p<0.05 is denoted by * as compared to control, # as compared to LPS alone.

Systemic exposure to LPS is known to result in a neuroinflammatory response [1,28–30]. Accordingly, exposure to LPS (2 mg/kg, s.c.) caused a time-dependent increase in expression of neuroinflammatory cytokines and chemokines in cortex (Fig 1A). Neuroinflammation was seen with a peak as early as 2 hours post-LPS. In some cases, neuroinflammation persisted to 12 hours after LPS exposure (e.g., TNFα and IL-1β). Overall, the response to LPS occurred throughout the brain (see Fig 2 below).

### Systemic exposure to LPS activates the STAT3 pathway without causing astrogliosis

IL-6, LIF, and OSM have been shown to bind to the gp130 receptor, with crosstalk input from the other cytokines/chemokines, and to subsequently activate the janus kinase 2 (JAK2) /STAT3 pathway (e.g., see O’Callaghan *et al*., 2014 [1]). Therefore, we evaluated the phosphorylation state of STAT3 at Tyr705 as an index of the downstream activation of the cytokine/JAK2/STAT3 signaling cascade. In agreement with our prior findings for effects of LPS in striatum [1], pSTAT3^Tyr705^ was significantly increased 6 hours after LPS exposure in all brain areas; cortex data are shown as representative of other areas (Fig 1B). This time point corresponds to the period after the onset of enhanced expression of cytokine and chemokine mRNA (Fig 1A), consistent with activation of STAT3 as a downstream effector of cytokine/chemokine signaling.

The close association of neuronal damage and neuroinflammation has made it difficult to separate the two conditions, with many publications inferring that neuroinflammation causes damage, or *vice versa*. Astrogliosis, as evidenced by an enhanced expression of GFAP, is a hallmark of toxicant-induced neuronal damage [31]. Previously, we have shown that neuroinflammation precedes the activation of the STAT3 pathway in astrocytes and the subsequent increase in expression of GFAP that is associated with toxicant-induced damage to dopaminergic nerve terminals [1]. In contrast, LPS-induced neuroinflammation, while activating the striatal STAT3 pathway, does not result in astrogliosis [1]. In agreement with these latter findings, LPS failed to increase GFAP in cortex (Fig 1C).

### Chronic exposure to CORT exacerbates LPS-induced neuroinflammation

**Fig 3:**
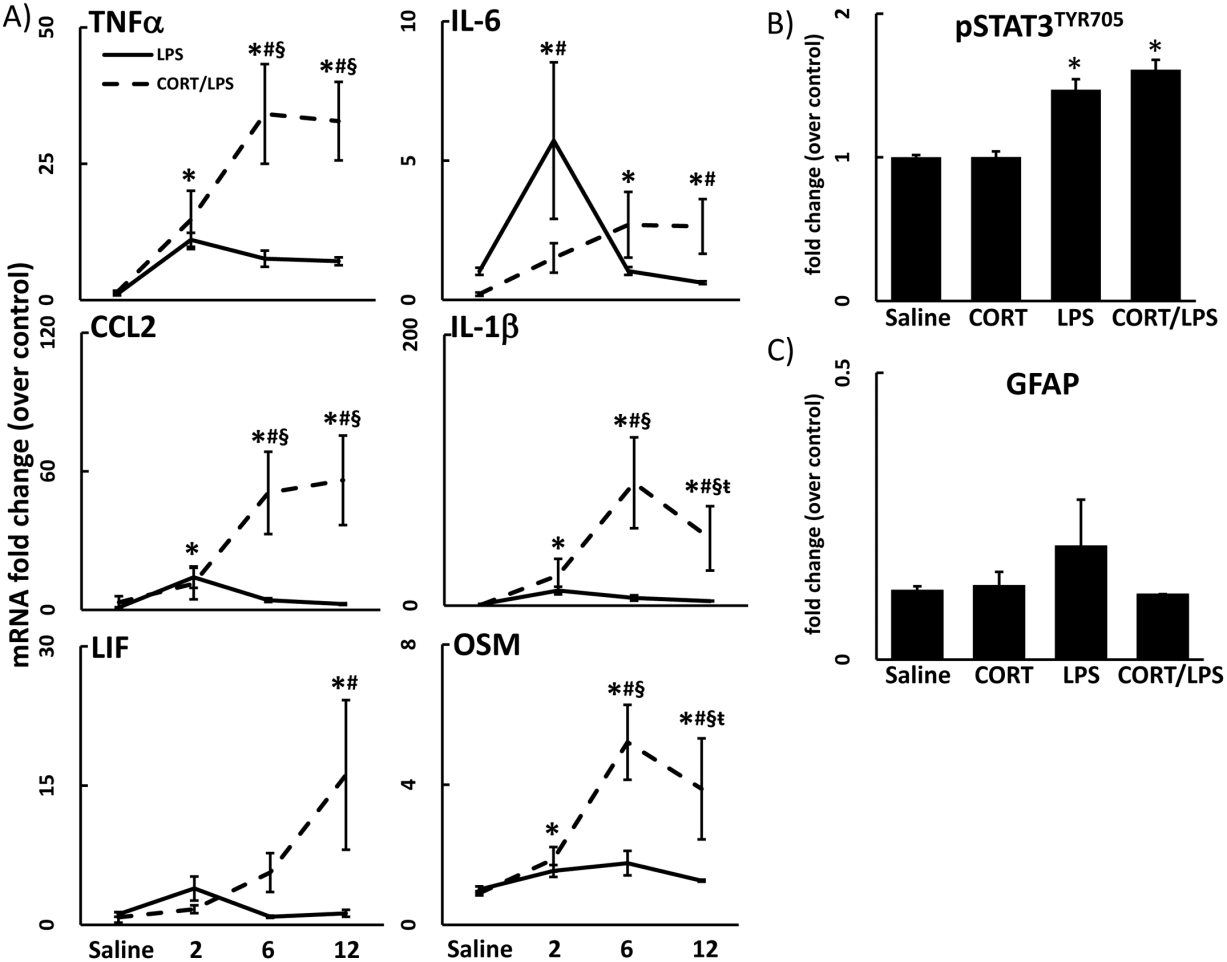
Chronic CORT pretreatment exacerbates and prolongs LPS-induced neuroinflammation but has no effect on activation of STAT3 and does not cause astrogliosis. A) A time course of LPS (2 mg/kg, s.c.)-induced neuroinflammation with and without CORT pretreatment (200 mg/L 0.6% EtOH in drinking water for one week) is shown (saline, 2, 6 and 12 hours). TNFα, IL-6, CCL2, IL-1β, LIF, and OSM mRNA levels were measured in samples prepared from cortex. Data represents mean ± SEM (n = 5 mice/group). CTX data for LPS alone treated groups are included from Fig 1A for statistical comparison. Statistical significance of at least p<0.05 is denoted by * as compared to control, # as compared to LPS alone, § as compared to 2h CORT/LPS, and ŧ as compared to 6h CORT/LPS. B & C) Effects of chronic CORT pretreatment (200 mg/L 0.6% EtOH in drinking water for one week) on LPS induced activation of the STAT3 signaling module was assessed by immunoblots of pSTAT3^Tyr705^ and astrogliosis was assessed by immunoassay of GFAP. Cortex samples were analyzed for pSTAT3^Tyr705^ (6 hours) and GFAP protein (72 hours) after LPS exposure of CORT pretreated animals. Data represent mean ± SEM (n = 5 mice/group). Saline and LPS alone treated groups are included from Fig 1B & 1C for statistical comparison. Statistical significance of at least p<0.05 is denoted by * as compared to control, # as compared to LPS alone.

Previously, we showed that week-long exposure to CORT enhanced both neuroinflammation and neurodegeneration in mice exposed to a neurotoxic regimen of methamphetamine [2]. Here, we again administered CORT in the drinking water for 1 week and found that the neuroinflammatory response to LPS was markedly exacerbated by CORT; this enhanced neuroinflammatory response to LPS occurred in several brain regions (Fig 2). The exacerbation of neuroinflammation induced by CORT pretreatment resulted in a longer lasting and more accentuated response as evidenced by significantly heightened neuroinflammation at 6 and 12 hours after LPS exposure in cortex (Fig 3A) compared to LPS exposure alone (LPS data from Fig 1A included for statistical comparison).

While prior CORT can enhance neurodegenerative responses seen with a neurotoxicant such as methamphetamine [2], an effect associated with increased activation of STAT3 and accompanying astrogliosis, these effects were not found with constant exposure to CORT in the drinking water prior to LPS (Fig 3B & 3C; saline and LPS data from Fig 1B & 1C included for statistical comparison). These observations are consistent with our prior data for LPS [1] where activation of STAT3 likely was not associated with activation of astrocytes, as we did not observe an accompanying increase in GFAP (Fig 3B; saline and LPS data from Fig 1B included for statistical comparison).

### Chronic exposure to CORT exacerbates neuroinflammation induced by another route of LPS exposure

**Fig 4:**
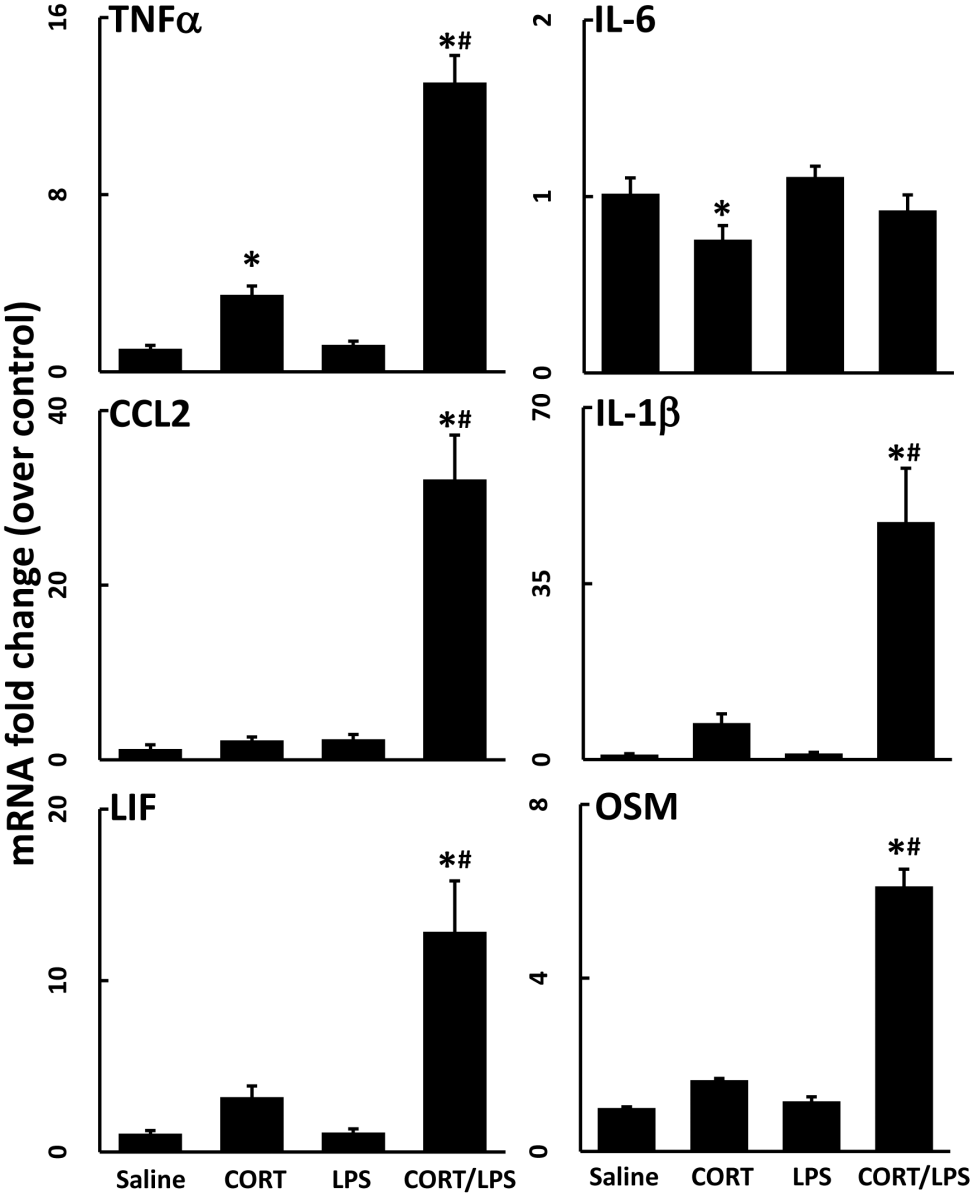
Chronic CORT sensitizes the CNS to a sub-neuroinflammatory inhalation dose of LPS. Mice pretreated with CORT (200 mg/L 0.6% EtOH in drinking water for one week) were subjected to 3 hours of LPS (7.5 μg/m^3^) by inhalation. TNFα, IL-6, CCL2, IL-1β, LIF, and OSM mRNA levels were measured in samples prepared from cortex collected immediately following LPS inhalation exposure. Data represent mean ± SEM (n = 5 mice/group). Statistical significance of at least P<0.05 is denoted by * as compared to respective control and # as compared to LPS alone exposed group.

Mice treated with CORT in the drinking water for 1 week were exposed to aerosolized LPS for 3 hours to determine if CORT affected the neuroinflammatory response to LPS via an alternate route of exposure. Inhalation exposure to LPS alone did not affect expression of neuroinflammatory cytokines, but the prior exposure to CORT resulted in a marked increase in all cytokines, except IL-6 (Fig 4). Interestingly, mice that inhaled aerosolized water after a week of CORT had a significantly elevated mRNA expression level of TNFα and slightly reduced IL-6 mRNA.

### Chronic exposure to CORT also exacerbates neuroinflammation induced by the inflammagen, PIC

**Fig 5:**
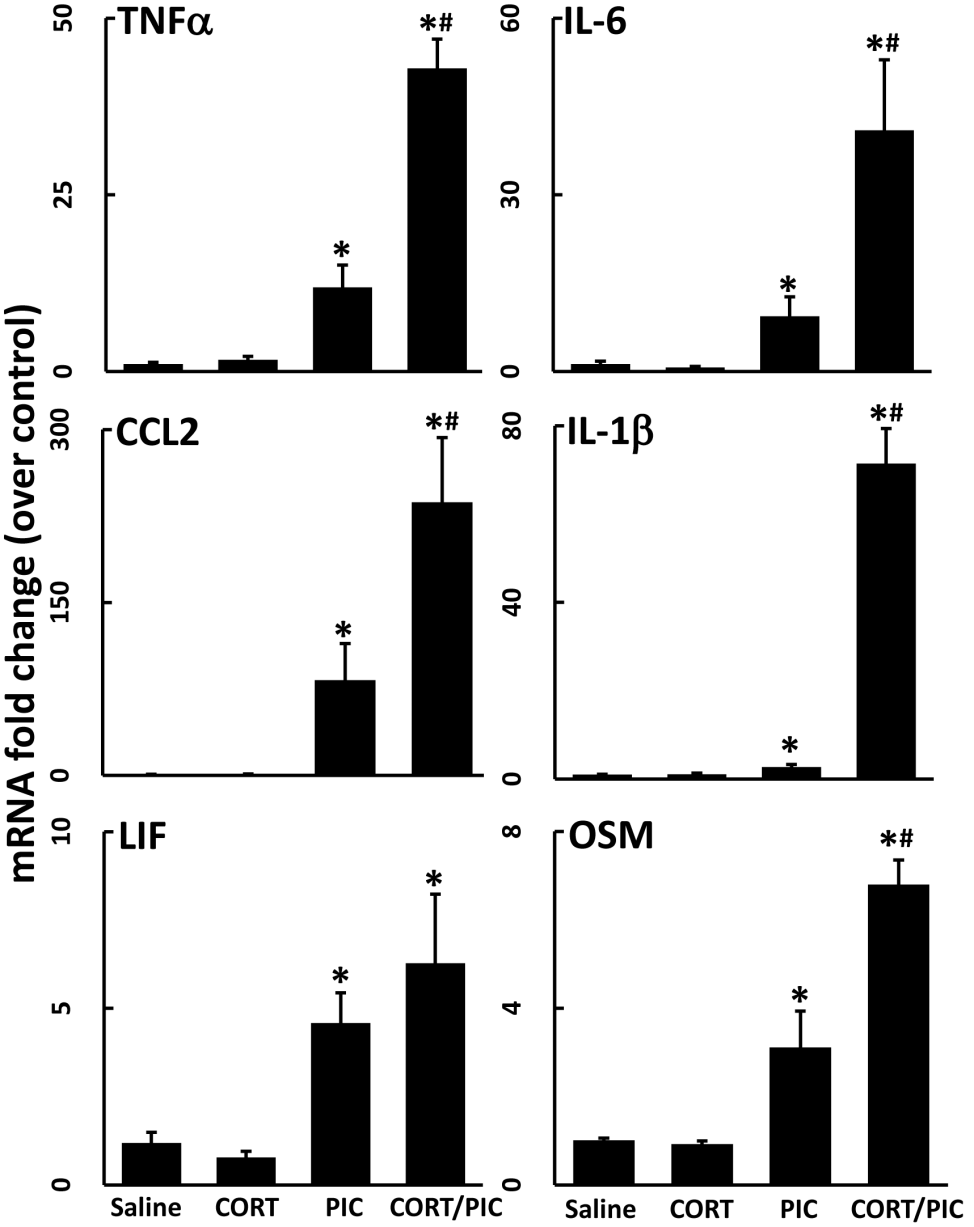
Chronic CORT exacerbates PIC-induced neuroinflammation. Mice were pretreated with CORT (200 mg/L 0.6% EtOH in drinking water for one week). TNFα, IL-1β, LIF, and OSM mRNA levels were measured in samples prepared from cortex collected 6 hours after PIC exposure (12 mg/kg, i.p.). Data represent mean ± SEM (n = 5 mice/group). Statistical significance of at least P<0.05 is denoted by * as compared to respective control group.

Similar to LPS, peripheral exposure to the viral mimic, PIC, is known to result in neuroinflammation throughout the brain [32–34]. As such, exposure to PIC alone caused a significant neuroinflammatory response at 6 hours post-exposure (Fig 5). Exposure to CORT in the drinking water for 7 days significantly exacerbated the PIC-induced expression of cytokines and chemokines (Fig 5).

### Single and recurring week-long exposures to CORT result in protracted neuroinflammatory priming

**Fig 6:**
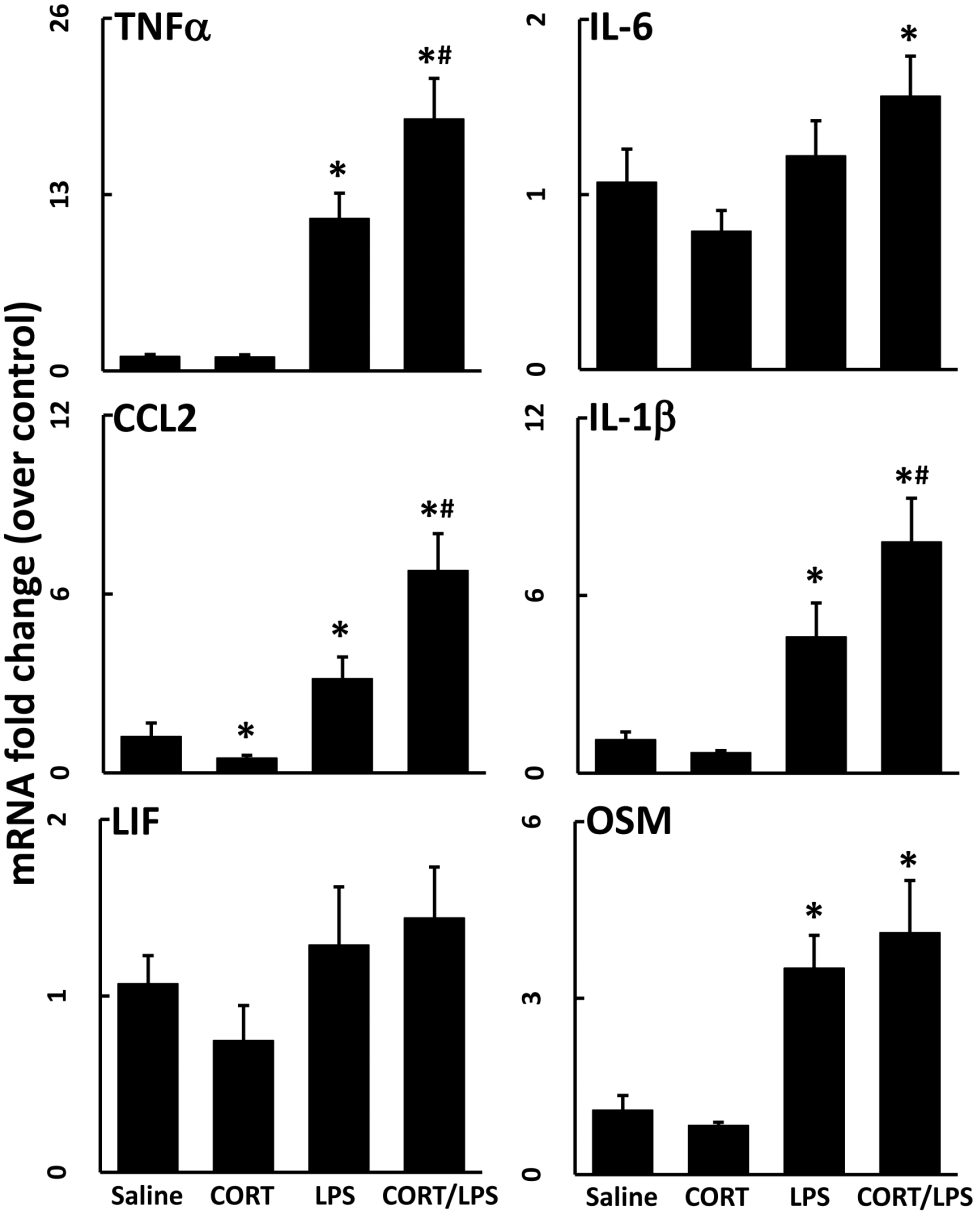
Chronic CORT pretreatment primes the neuroinflammatory response for 30 days. Mice were pretreated with CORT (200 mg/L 0.6% EtOH in drinking water for one week). Thirty days after the cessation of CORT, mice were administered LPS (2 mg/kg, s.c.). TNFα, IL-6, CCL2, IL-1β, LIF, and OSM mRNA levels were measured in samples prepared from cortex 6 hours after LPS exposure. Data represent mean ± SEM (n = 5 mice/group). Statistical significance of at least P<0.05 is denoted by * as compared to appropriate control and # as compared to LPS alone.

**Fig 7:**
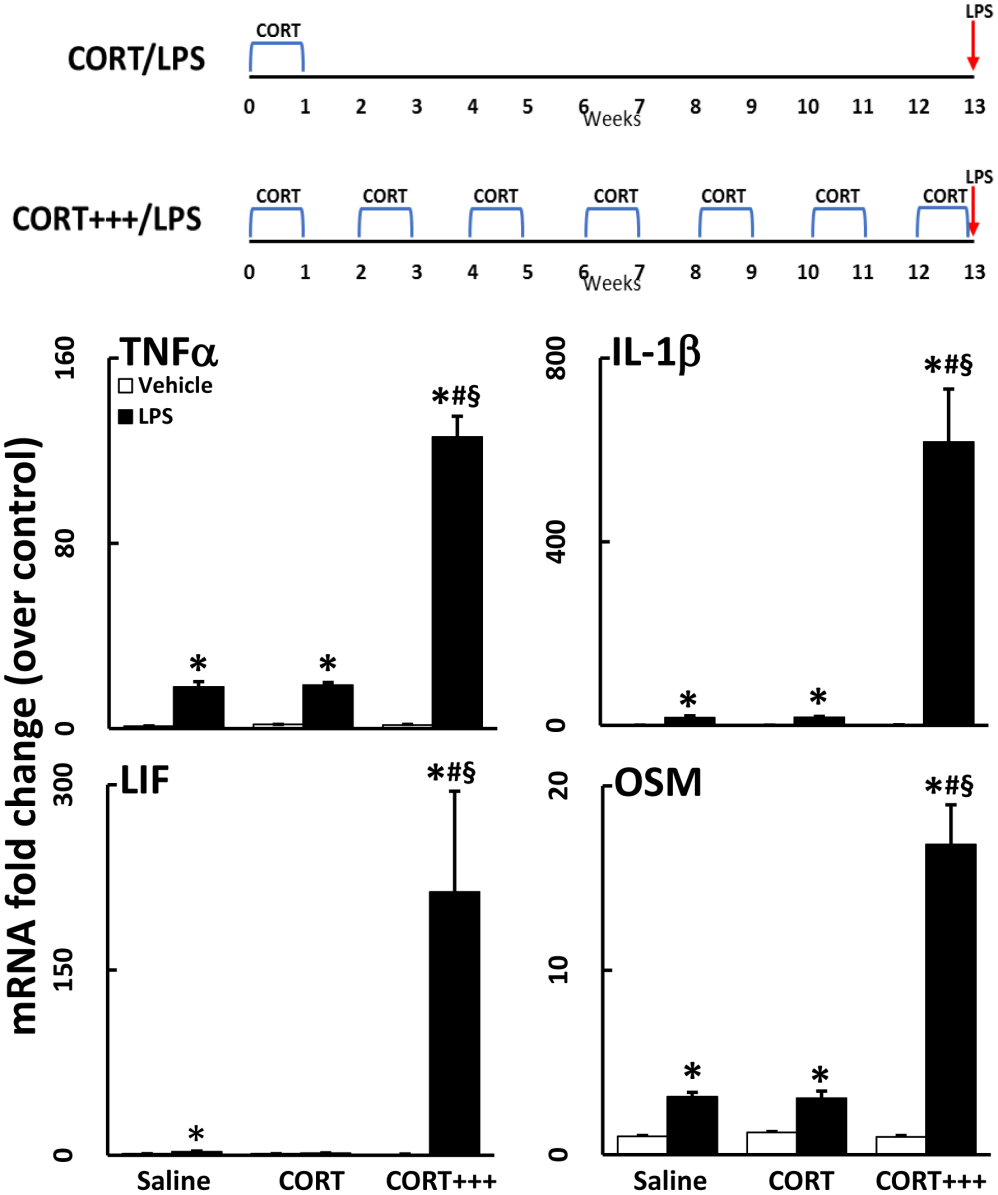
Successive waves of CORT for 90 days greatly exacerbates LPS-induced neuroinflammation. Mice were treated with CORT (200 mg/L 0.6% EtOH) for 7 days in the drinking water followed either by 90 days without (CORT) or 90 days of bimonthly, 7 day CORT exposures (CORT+++). LPS (2 mg/kg, s.c.) was administered on day 97. TNFα, IL-1β, LIF, and OSM mRNA levels were measured in samples prepared from cortex collected 6 hours after LPS exposure. Data represent mean ± SEM (n = 5 mice/group). Statistical significance of at least P<0.05 is denoted by * as compared to appropriate control, # as compared to LPS alone, and § as compared to CORT/LPS.

While we have shown that a week-long exposure to high physiological levels of CORT in the drinking water serves to potentiate the neuroinflammatory response to systemic LPS administration, the persistence and duration of this effect are unknown. To further define the effects of CORT priming on the neuroinflammatory response to LPS, mice were exposed to CORT in the drinking water for 1 week, but then not exposed to LPS until 30 days later. Even 30 days after the cessation of exposure to CORT, the neuroinflammatory response remained primed to exacerbate LPS-induced neuroinflammation (TNFα, CCL2, IL-1β) (Fig 6)(compare magnitude of mRNA changes in this figure with those shown in Fig 3). However, an exacerbated neuroinflammatory response to LPS had dissipated by 90 days (Fig 7; CORT/LPS) after CORT treatment, as evidenced by the similarity in mRNA expression between CORT/LPS and LPS alone treated groups. When mice were exposed to multiple, successive waves of 1-week exposures to CORT over 90 days, however, the neuroinflammatory response to LPS was potentiated up to 10-fold greater than the response after a single 1-week CORT exposure prior to LPS (Fig 7; CORT+++/LPS).

## Discussion

We have demonstrated that exposure to CORT in the drinking water, at levels designed to mimic high physiological stress levels of this hormone, markedly enhances the neuroinflammatory response to LPS and the viral mimic, PIC. This neuroinflammatory “priming” by CORT is so effective that previously sub-neuroinflammatory exposures to LPS by inhalation instigate significant neuroinflammation when combined with prior exposure to CORT. Furthermore, a single week of exposure to CORT in the drinking water maintains the potential for priming the neuroinflammatory response to LPS for 30 days. Most remarkably, intermittent exposure to 7 days of CORT for up to 90 days serves to markedly escalate the LPS-induced neuroinflammatory response.

Neuroinflammation initiated by both neural damage and LPS is propagated through the STAT3 signaling cascade [1,28]. However, while CORT primed the LPS-induced neuroinflammation, we found that the activation of STAT3 (pSTAT3^Tyr705^) was unaffected by prior CORT exposure (Fig 3B). STAT3 activation due to neural damage is associated with astrogliosis and subsequent elevations in the expression of the astrocyte intermediate filament protein, GFAP. STAT3 activation caused by inflammagens such as LPS is not necessarily associated with neural damage [1] and, therefore, is not accompanied by an increase in GFAP (Figs 1 and 3). Thus, the lack of an increase in pSTAT3^Tyr705^ or GFAP with CORT given prior to LPS suggests that the CORT-induced exacerbation of LPS-induced neuroinflammation is not accompanied by neural damage. The multiple cytokine pathways affected implicate involvement of microglia, to be explored in the future by more detailed analyses of components of the inflammasome.

Previous studies have demonstrated a glucocorticoid-induced enhancement of neuroinflammation. An investigation that used CORT in the drinking water for a longer duration (10 days vs. the 7 days used here) evaluated the response of cultured primary microglia to LPS exposure and found that microglia secreted more IL-1β, IL-6, and TNFα with longer CORT pretreatment [17]. Using CORT pellets to mimic mild stress, adult adrenalectomized rats also exhibited increased brain expression of TNFα and IL-1β, along with NF-κB activation [18]. In a model of neuropathic pain, intrathecal administration of LPS 24 hours following a single subcutaneous injection of CORT resulted in exacerbated LPS-induced allodynia and increased expression of IL-1β and IL-6 in the spinal cord [19]. These studies combined with the work presented here illustrate that CORT exposure has the potential to exacerbate LPS-induced neuroinflammation both acutely and for a prolonged period (i.e., for 1–3 months, Figs 6 and 7). We have expanded upon the results of previous studies by broadening the number of neuroinflammatory cytokines and chemokines examined, as well as by showing the altered kinetic expression profiles of these genes following CORT pretreatment as well as the brain-wide nature of the CORT-primed neuroinflammatory response. Additionally, our findings appear to be the first to show an escalating priming effect on neuroinflammation at long-term time points after episodic CORT exposures.

While chronic CORT exposure was used in this study to mimic high levels of physiological stress, several others have shown that behavioral stress paradigms can also be pro-inflammatory. For example, a 2 hour repeated social defeat paradigm used in rats increased plasma IL-1β levels in response to LPS administration [14]. Gibb *et al*. [35] found that both acute (social disruption) or chronic (rotating stressor) stress paradigms prior to systemic LPS exposure resulted in protracted sickness behavior and increased expression of cytokine mRNA in the brain. Evaluation of these studies reveals that the repeated social defeat protocol used by Carobrez and colleagues [14] and the chronic stress paradigm used by Gibb and colleagues [35] result in a greater increase in plasma CORT compared to other paradigms that were not found to be proinflammatory [15,16]. These observations combined with our results clearly indicate that the conditions of CORT exposure are a crucial factor in determining its pro-inflammatory consequences. Furthermore, the variability of the responses to inflammatory challenge following stressor paradigms highlights the utility of our exogenous CORT exposure regimen, which should, theoretically, produce a more uniform stress hormone response.

LPS stimulates the bacterial acute phase response of innate immunity, which is characterized by the release of inflammatory cytokines. Similarly, the synthetic dsRNA PIC instigates the viral acute phase response. As the results of combining a stressor with PIC are mixed [36–38], we exposed CORT-treated mice to PIC to test whether the CORT priming effect was generalizable to another inflammagen. Similar to LPS, the neuroinflammatory response instigated by peripheral PIC exposure was exacerbated by prior exposure to CORT (Fig 5). This is particularly interesting because LPS and PIC have slightly different effects when administered peripherally. First, LPS is a lipophilic compound that readily enters the bloodstream, gaining access to the CNS to activate central neuroinflammation by binding to LPS receptors in the brain [39]. However, intraperitoneally-administered PIC does not enter blood circulation and, conversely, instigates neuroinflammation through peripherally-generated inflammatory mediators [40]. While it is possible that the mechanisms by which CORT pretreatment affects the neuroinflammatory response to these two inflammagens may diverge, it is likely that CORT exposure acts upon the overlapping signaling mechanisms induced by the peripherally-generated cytokines and chemokines following LPS or PIC exposure. This could be achieved through modulation of neural cytokine/chemokine receptors or changes in the transport of peripheral inflammatory mediators across the blood brain barrier by CORT. Second, LPS and PIC signal through different receptors, with LPS signaling through Toll-like receptor 4 (TLR4) and PIC signaling through Toll-like receptor 3 (TLR3), retinoic acid-inducible gene 1 (RIG1), and melanoma differentiation-associated protein 5 (MDA5). The convergence on toll-like receptor signaling between both of these inflammagens makes TLR signaling a viable candidate for further investigation into how CORT exposure instigates inflammatory priming. This may result from effects on the LPS and PIC receptors directly, or indirectly through modulation of shared aspects of the inflammatory cytokine expression profiles instigated by these inflammagens. Overall, the observation that CORT exacerbates LPS-induced and PIC-induced neuroinflammation, as well as our prior observations with neurotoxicant exposures [1–4], helps to build a library of disparate inflammatory conditions comprised of numerous signaling cascades which can be utilized and manipulated to discover a convergent mechanism by which CORT exposure primes neuroinflammation.

## Conclusion

Our findings confirm that prior exposure to CORT resembles the effects of behavioral stressors, several of which markedly increase neuroinflammation associated with a subsequent innate immune system challenge with LPS. When this week-long prior exposure to CORT is extended to multiple weekly episodes of CORT over three months, a dramatic escalation of the neuroinflammatory response to LPS is seen, suggestive of the potential for chronic stressors to cause long-lasting and severe neuroinflammation. Neuroinflammatory signaling serves as the molecular basis for sickness behavior, symptoms of which are associated with chronic neurological disease states such as depression, chemobrain, neuropathic pain and Gulf War Illness [3,4,41–43]. We conclude that there is the potential for recurrent physiological stressors to contribute to and greatly exacerbate such chronic inflammatory disorders.

## Abbreviations

CB: Cerebellum
CCL2: Chemokine (C-C motif) Ligand 2
CORT: Corticosterone
CTX: Cortex
GAPDH: Glyceraldehyde-3-phosphate dehydrogenase
GFAP: Glial Fibrillary Acidic Protein
HIP: Hippocampus
HYPO: Hypothalamus
IL-1β: Interleukin 1 beta
IL-6: Interleukin 6
JAK2: Janus Kinase 2
LIF: Leukemia Inhibitor Factor
LPS: Lipopolysaccharide
OB: Olfactory Bulb
OSM: Oncostatin M
PIC: Polyinosinic:Polycytidylic Acid
s.c.: Sub cutaneous
SDS: Sodium Dodecyl Sulfate
STAT3: Signal Transducer and Activator of Transcription 3
STR: Striatum
TLR3/TLR4: Toll-like Receptor 3 or 4
TNFα: Tumor Necrosis Factor Alpha
